# Immunomodulatory Properties of Polysaccharide-Rich Young Green Barley (*Hordeum vulgare*) Extract and Its Structural Characterization

**DOI:** 10.3390/molecules27051742

**Published:** 2022-03-07

**Authors:** Marta Kinga Lemieszek, Iwona Komaniecka, Michał Chojnacki, Adam Choma, Wojciech Rzeski

**Affiliations:** 1 Department of Medical Biology, Institute of Rural Health, Jaczewskiego 2, 20-090 Lublin, Poland; chojnacki.michal@imw.lublin.pl (M.C.); rzeskiw@hektor.umcs.lublin.pl (W.R.); 2 Department of Genetics and Microbiology, Institute of Biological Sciences, Maria Curie-Skłodowska University, Akademicka 19, 20-033 Lublin, Poland; iwona.komaniecka@poczta.umcs.lublin.pl (I.K.); adam.choma@mail.umcs.pl (A.C.); 3 Department of Functional Anatomy and Cytobiology, Institute of Biological Sciences, Maria Curie-Skłodowska University, Akademicka 19, 20-033 Lublin, Poland

**Keywords:** cancer immunotherapy, fructan, FT-IR, mass spectrometry, young green barley

## Abstract

Young green barley (YGB) water extract has revealed a beneficial impact on natural killer (NK) cells’ ability to recognize and eliminate human colon cancer cells, without any side effects for normal colon epithelial cells. The direct anticancer effect of the tested compounds has been also shown. The mixture of oligosaccharides found in this extract was characterized by chemical analyses and via FT-IR spectroscopy and MALDI-TOF MS techniques. The YGB preparation contained 26.9% of proteins and 64.2% of sugars, mostly glucose (54.7%) and fructose (42.7%), with a small amount of mannose (2.6%) and galactose (less than 0.5%). Mass spectrometry analysis of YGB has shown that fructose oligomers contained from 3 to 19 sugar units. The number of fructans was estimated to be about 10.2% of the dry weight basis of YGB. The presented results suggest the beneficial effect of the consumption of preparations based on young barley on the human body, in the field of colon cancer prevention.

## 1. Introduction

Oncological immunotherapy, also known as immuno-oncology, is a modern method of cancer treatment consisting of administering non-toxic preparations, modulating the immune system’s response by increasing and/or improving its ability to prevent, control, and fight cancer. Immuno-oncology can educate the immune system to better recognize, more effectively attack, and successfully eliminate cancer cells. At the beginning of the 21st century, the American Society of Clinical Oncology announced that oncological immunotherapy will dominate the treatment of neoplastic diseases and, after 2020, it could be used in the treatment of even every second type of cancer. In the beginning, immuno-oncology mainly focused on immunostimulatory cytokine treatments. Recently, natural killer cell-based immunotherapy has emerged as the most promising therapeutic approach for both hematological malignancies and solid tumors [1]. The natural killer (NK) cells were first identified as unique large granular lymphocytes able to detect and rapidly kill abnormal cells without prior sensitization; thus, they are the first line of defense for the immune system against cells infected with bacteria and viruses, as well as malignant cells [2]. The importance of NK cells for cancer treatment has been confirmed in epidemiological studies, which revealed the negative correlation between NK cells activity and the risk of cancer development and metastasis [1]. The main considerations relating to NK cell-based immunotherapy are the NK cell source and the method of enhancement of NK cell anticancer activity. The presented studies address the second issue, in particular, being focused on barley (*Hordeum vulgare*), which is the fourth most important grain crop in the world and the richest source of dietary fiber among the cereals. For many years, barley was valued solely for its nutritional properties; recently, more and more scientific reports have indicated its health benefits. In the early 1990s, Japanese scientists showed that the most valuable source of both nutrients and bioactive substances is young green barley, defined as seedlings up to 200 h after sprouting, with a height of between 20 and 30 cm [3,4,5]. More recent studies have also indicated barley grass as a functional food with many health-promoting effects (e.g., anti-aging, antioxidant, anti-inflammatory, immunity enhancement, detoxification, hypolipidemic effects, improvement of gastrointestinal activity, blood pressure regulation, anti-hypoxia, antifatigue) as well as a potential agent against several human chronic diseases, including cancer, cardiovascular disease, obesity, diabetes, and stroke [6]. The health benefits of young green barley are associated with many of its different elements; however, immunomodulatory properties are typically considered to be due to providing a large amount of dietary fiber, especially in the form of polysaccharides [6,7,8]. The immunoenhancement effects of polysaccharides isolated from young barley leaves have been demonstrated by Kim et al. and Han et al., in both in vitro and in vivo studies [9,10]. Kim et al. have reported the stimulation of proliferation and the production of cytokines (IL-6, GM-CSF) in cells derived from Peyer’s patches, in response to arabinoxylan- and rhamnogalacturonan I-rich polysaccharides that were isolated from barley grass (BLE0) [9]. Furthermore, the intestinal immunostimulatory activity observed in vitro has been also proved in mice, wherein the oral administration of BLE0 augmented IgA production and increased the levels of IgA-related cytokines (IL-10, TGF-β) [9]. Further studies on the abovementioned crude barley polysaccharides (BLE0) confirmed their immunostimulatory effects. They have been shown to elicit an increase in the proliferation and secretion of cytokines (IL-2, IL-4, IL-10, IFN-γ) in CD3/CD28-activated mice splenocytes. Additionally, oral administration of barley polysaccharides enhanced the immune system of mice with cyclophosphamide-induced immunosuppression, via the stimulation of splenocyte proliferation and acceleration of NK cell cytotoxic activity [10]. Han et al. have shown that water-soluble barley polysaccharide (BP-1) significantly improved the immune ability of immunosuppressive mice in several different ways, including: increasing the number of bone marrow cells as well as white blood cells in peripheral blood; promoting the proliferation of spleen cells, NK cells and macrophages; stimulating NK cell cytotoxic activity; improving the phagocytosis activity of macrophages; increasing the level of IL-2, TNF-α and IFN-γ in serum and the spleen; and enhancing the production of IgG and IgM in the spleen [11]. Ryu et al. have shown that water-soluble barley β-glucan suppressed the proliferation of mice splenocytes, accelerated by concanavalin A, IL-2, or alloantigen. Additionally, the tested β-glucan suppressed the death-induced activity of allogenic lymphocytes Tc and allogenic lymphokine-activated killer cells [12]. These immunomodulatory properties were also reported in the case of commercially available barley β-glucan. Misra et al. demonstrated that β-glucan derived from barley improved immune system activity in fish through an increase in leukocyte number, complemented activation, and stimulated phagocytic activity as well as bactericidal activity [13]. Aoe et al., who investigated the impact of the high-fructan barley product named BARLEYmax (BM; containing fructan 9.0%, β-glucan 6.3%, and resistant starch 3%) on the growth of microbiota that are present in the rat gut, revealed a beneficial effect of the investigated carbohydrates on health-promoting species belonging to the *Bifidobacterium*, *Oscillospira*, *Parabacteroides* and *Sutterella* species, which may prevent colonic inflammation and enhance intestinal immunological functions [14]. Furthermore, Aoe et al. have shown that BM possesses stronger prebiotic properties than the high-β-glucan barley line (BG; containing β-glucan 9.0%, fructan, 2.2%, and resistant starch, 0.5%) [14]. The beneficial effect of BARLEYmax on the gut microbiome was also proven in clinical studies, wherein 4 weeks’ consumption at 12 g/day of this high-fructan barley product increased the abundance of *Bacteroides* and decreased the abundance of *Clostridium subcluster* [15]. Similarly, Sasaki et al. revealed in an in vitro model of the human colonic microbiota that polysaccharide-rich young green barley leaf extract (YBL; containing carbohydrates at 46.5%, including fructan at 9.4%, proteins at 29.0%, and minerals at 17.5%) increased the abundance of bacteria related to the genus *Bifidobacterium* and stimulated the growth of bacteria related to the genera *Faecalibacterium, Lachnospira*, *Roseburia,* and unclassified *Ruminococcaceae*. The discovered bifidogenic and butyrogenic effects of YBL have been associated with health-promoting properties as a result of gut immune system stimulation, as well as via inflammatory inhibition [16].

Despite the fact that several scientific reports have revealed the immunoenhancement properties of polysaccharides derived from barley, due to the high variability of polysaccharides and the associated diversity of biological activities, it is worth continuing research into this group of compounds. The aim of the current study was to evaluate the immunomodulatory properties of the water extract of young green barley (*Hordeum vulgare*) reached with polysaccharides by evaluating its influence on the viability and proliferation of NK-92 cells and above all their ability to kill human colon cancer LS180 cells representative for the common cancers worldwide. At the same time, the selectivity of the anticancer effect of polysaccharide-activated NK cells was also evaluated on the human normal colon epithelial CCD841 CoN cells. Furthermore, to investigate the major compounds related to the immunomodulatory effect, the structural characterization of extract polysaccharides was conducted.

## 2. Results

### 2.1. Young Green Barley Extract Composition

**Figure 1:**
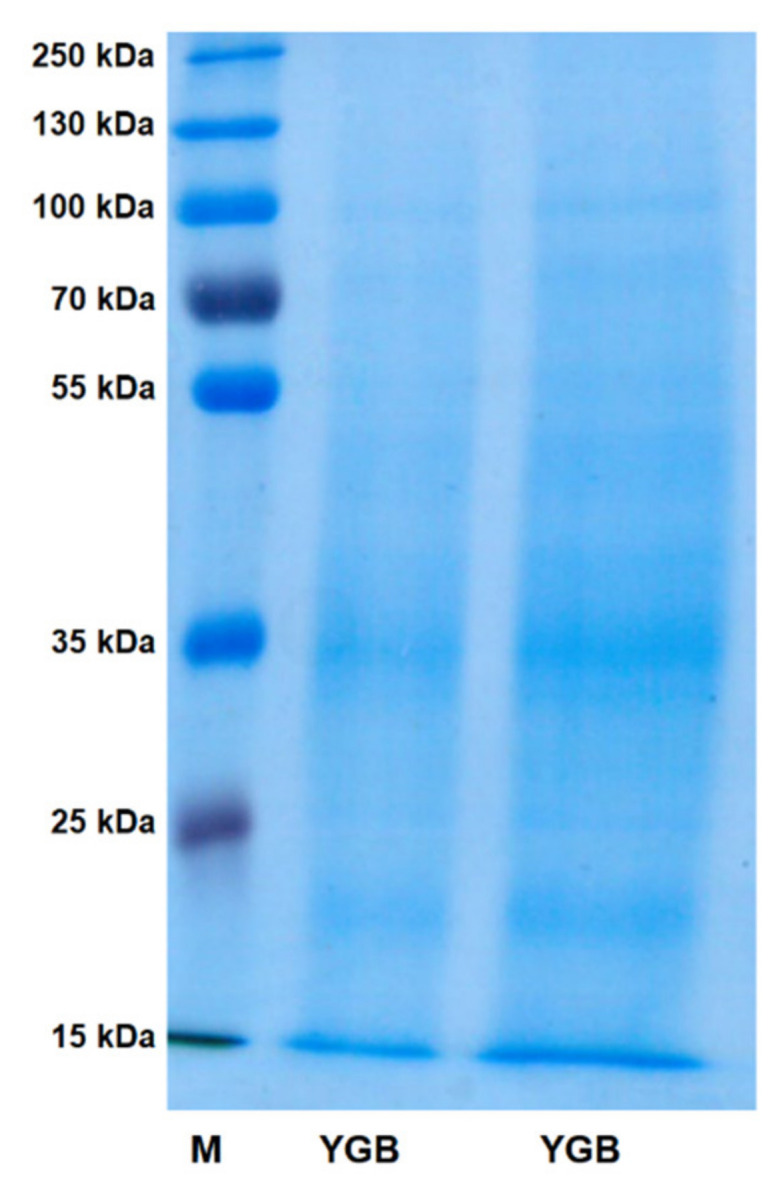
Electrophoretic separation (SDS-PAGE) of young green barley extract (YGB). The 12% polyacrylamide gel used after protein separation was stained with Coomassie Brilliant Blue. As a standard, a molecular protein mass marker (from 15 to 250 kDa) was used.

In the first stage, the chemical composition of young green barley extract was investigated. The total sugar and protein contents were examined using colorimetric methods (phenol-sulphuric acid assay, Pierce BCA protein assay). These studies revealed that the tested extract consisted mainly of sugars (64.2 ± 1.14%), while the protein amount (26.9 ± 4.09%) was also significant. Electrophoretic separation (SDS-PAGE) of young green barley extract (YGB) confirmed the presence of proteins with a molecular weight of around 55 kDa and 35 kDa, as well as proteins weighing less than or equal to 15 kDa. The obtained data could indicate that YGB is a carbohydrate-protein complex (Figure 1).

**Figure 2:**
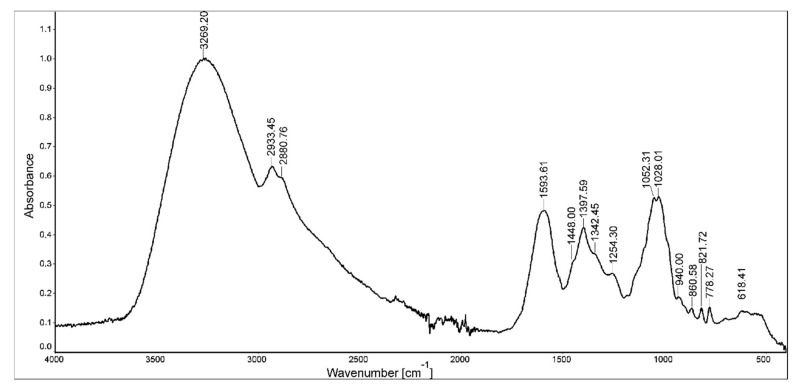
FTIR spectrum of young green barley extract (YGB).

The FT-IR spectrum of YGB preparation ranged from 400 cm^−1^ to 4000 cm^−1^, as shown in Figure 2. The results showed an intense and broad absorption peak at 3269 cm^−1^ for O-H and N-H stretching vibrations, a peak at 2933 cm^−1^ for C-H stretching vibrations, and a broad absorption band in the region of 950–1200 cm^−1^, with a maximum at 1053 for coupled C-O and C-C stretching and C-OH bending vibrations. All these absorption bands are characteristic of polysaccharides [17]. The peaks at around 2933 cm^−1^ and 2880 cm^−1^ indicate the presence of C-H bonds (C-H stretching vibration of CH_3_ and CH_2_ groups from carbohydrate molecules, respectively) [18]. The broad symmetrical signal (ranging from 1740 to 1500 cm^−1^), with a maximum at 1593 cm^−1^, might be indicative of proteins as well as adsorbed water (bending vibrations from amide I and amide II; absorbing groups: C=O, C-N, N-H, and H-O-H) [19,20]. The next intense broad signal at 1397 cm^−1^ corresponded with the absorbance of the CH_2_ and CH_3_ functional groups (deformational vibrations) from both sugars and proteins. The prominent and overlapping absorption bands between 1150 and 970 cm^−1^ were assigned to the C-O-C glycosidic bond vibrations and ring vibrations, as well as the C-O-H stretching vibrations of side-group bounds. These are commonly present in sugars [21]. The YGB preparation also showed absorption peaks at 940 and 860 cm^−1^, which are especially characteristic of α-d-glucose [22]. A broad signal at 618 cm^−1^ could be indicative of pyranose rings [23]. A very intense band with a maximum at ~1050 cm^−1^, and two sharp lines of similar intensity in the regions of 820 and 780 cm^−1^ indicate the presence of fructose from fructans [18].

Standard sugar analysis of the YGB preparation revealed the presence of glucose (87%), and small amounts of mannose and galactose (7% and 6%, respectively). This method does not allow the detection of ketoses (which are, in this case, converted to two alditol acetates). Therefore, YEB preparation was hydrolyzed under mild conditions (1 M HCl_aq_, 1 h, 50 °C), and the liberated monosugars were converted into methoxime peracetate (MOA) derivatives [24,25]. This procedure allowed us to identify fructose (42.7%) in an amount comparable to glucose (54.7%). A small amount of mannose (2.6%) and only traces of galactose (less than 0.5%) were also detected.

Table 1 contains the results of the methylation analysis and shows all derivatized compounds that were found. Fructose derivatives were found as terminal, →6)-linked and branched, →1,6)-linked forms, whereas glucose was terminal, →3)-linked, →4)-linked, and →6)-linked, as well as branched, →3,6)-linked. Mannose was exclusively terminal, and galactose—terminal, →3)-linked and →6)-linked. Among all the above-mentioned derivatives, terminal sugars prevailed, giving about 75% of all methylated end-products.

For the determination of fructan content, the PAHBAH reducing sugar method was used. The fructan content of young barley was calculated from the absorbance of the color complex and was then converted to a dry weight basis per 100 g of product. The average fructan content of the tested samples was 10.23 ± 0.22 g per 100 g dry weight basis (i.e., 10.2% of dry weight YEB extract).

**Figure 3:**
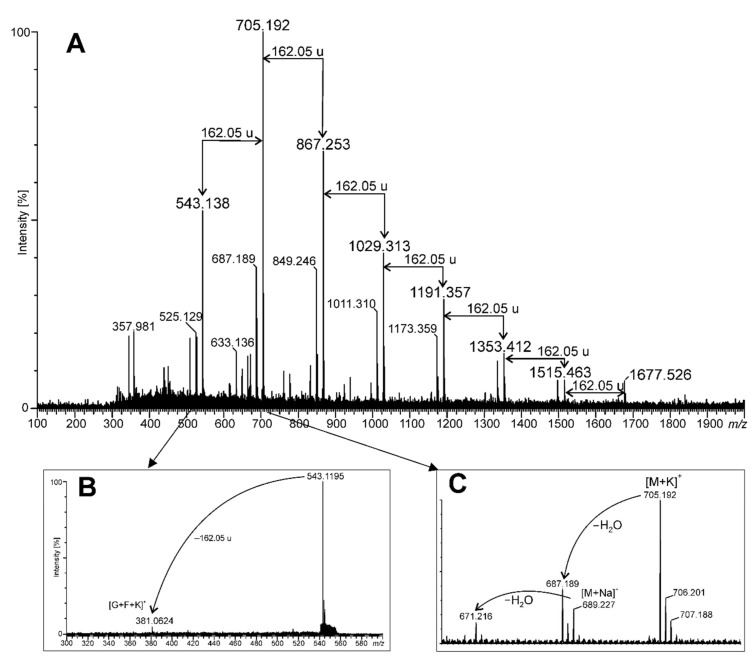
MALDI-TOF mass spectrum of young green barley extract (YGB) obtained in positive ion mode (**A**); MS-MS of the ion at *m/z* 543.1 (**B**); details of the spectrum A in the range of 670–710 *m/z* (**C**).

The size of the oligomer/polymer of carbohydrates present in YEB was estimated using the mass spectrometry technique. The MALDI-TOF mass spectrum, in positive ion mode, is shown in Figure 3. A series of intensive signals appeared in the same intervals of 162.05 u, pointing to hexose as a repeating unit. In the MALDI-TOF mass spectrum of YEB (Figure 3A), only those polymers containing up to 10 hexose units (DP = 10) were shown, but, when tracing the spectrum at low intensities in the mass range from 1700 to 3000 *m/z*, it was possible to find oligomers up to DP = 19 (data not shown). The smallest oligomer visible in Figure 3A had *m/z* at 543.138. Its fragmentation pattern has been shown in Figure 3B. This MALDI MS-MS spectrum revealed the only fragment ion *m/z* at 381.062, which corresponded to a disaccharide potassium adduct, probably composed of glucose and fructose residues (sucrose, [G+F+K]^+^). This disaccharide may be here a kind of backbone to which the next fructose units are linked during the biosynthesis process, forming the fructan detected in the YGB preparation.

The complexity of the MALDI-TOF mass spectrum is displayed in Figure 3C, showing that the main signal (oligomer potassium adduct, [M+K]^+^) is accompanied by the sodium adduct ([M+Na]^+^), as well as these molecules that are deprived of water ([M-H_2_O+K]^+^ and [M-H_2_O+Na]^+^, respectively). It should be noted that the protonated ([M+H]^+^) ions were not detected at the mass spectrum; thus, it can be concluded that the YGB preparation is a rich source of potassium and sodium salts.

### 2.2. Young Green Barley Extract Impacts NK-92 Cell Viability and Proliferation

**Figure 4:**
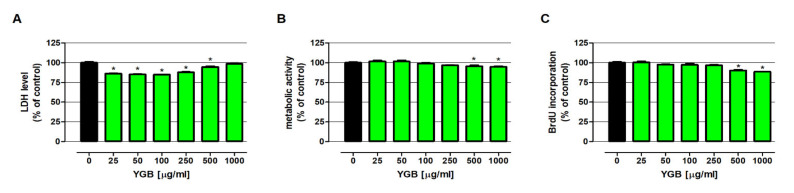
The influence of young green barley extract (YGB) on NK-92 cell viability and proliferation. Cells were exposed to culture medium alone (control) or with YGB at the following concentrations of 25, 50, 100, 250, 500, and 1000 µg/mL for 48 h. Cell viability was determined by the examination of both cell membrane integrity, using an LDH assay (**A**), and metabolic activity using an MTT assay (**B**), while cell proliferation was measured via an immunoassay based on BrdU incorporation (**C**). The results are presented as a mean ± SEM of at least 4 measurements. * *p* < 0.05 vs. control, one-way ANOVA test; post hoc test: Dunnett.

In order to evaluate the immunomodulatory potential of the YGB, its influence on NK-92 cell membrane integrity, metabolic activity, and proliferation was assessed via the LDH (lactate dehydrogenase), MTT (thiazolyl blue tetrazolium bromide), and BrdU (bromodeoxyuridine) tests, respectively (Figure 4). The LDH test revealed that the tested extract was not cytotoxic against NK-92 cells. Furthermore, the extract, when in concentrations from 25 µg/mL to 500 µg/mL, significantly decreased the lactate dehydrogenase release from treated cells. The MTT test has shown that YGB, when in concentrations from 25 µg/mL to 250 µg/mL, did not impact NK-92 viability, while at concentrations of 500 and 1000 µg/mL, it decreased the lymphocytes’ metabolic activity by 4.4% and 5.1%, respectively. The BrdU test revealed no changes in the proliferation of NK-92 cells treated with YGB when used in concentrations equal to or less than 250 µg/mL. On the contrary, the tested extract, when used at concentrations of 500 and 1000 µg/mL, inhibited DNA synthesis in NK-92 cells, reducing cell proliferation by 9.8% and 11.4%, respectively.

### 2.3. Young Green Barley Extract Enhances NK-92 Cells’ Cytotoxicity in Human Colon Cancer LS180 Cells without any Side Effects on Human Colon Epithelial CCD841 CoN Cells

**Figure 5:**
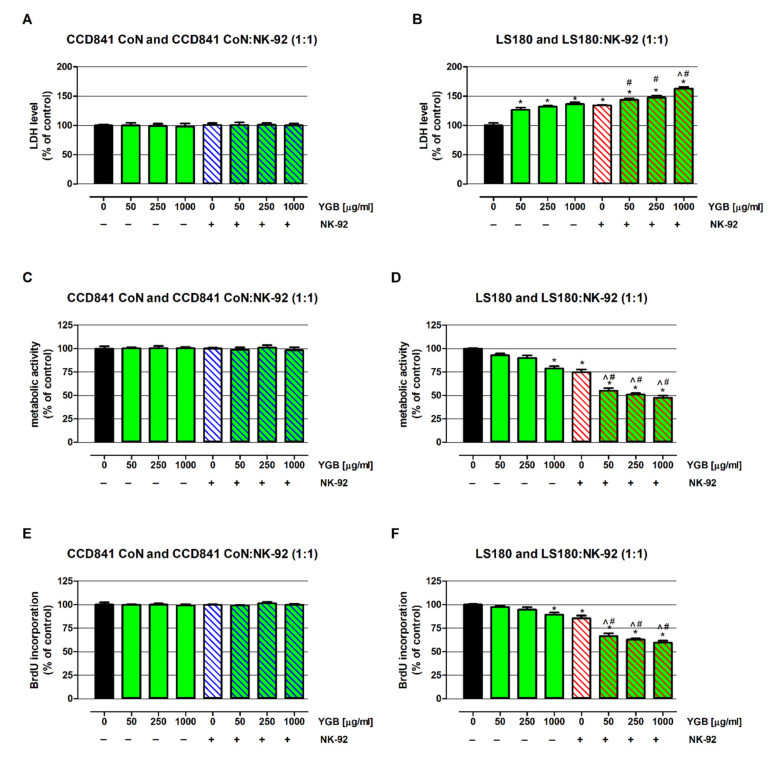
Influence of young green barley extract (YGB) on NK-92 cell cytotoxicity against normal and cancer colon cells. The human colon adenocarcinoma cell line LS180 and human colon epithelial cell line CCD841 CoN, and were exposed to culture medium alone (control) or the YGB (50, 250, and 1000 µg/mL) used alone, or to NK-92 cells in the absence or presence of YGB (50, 250 and 1000 µg/mL) for 48 h. NK-92 cells were used in the same concentration as treated cells. Changes in investigated cells in response to YGB, used alone or together with NK cells, were determined by examining cell membrane integrity using an LDH assay (**A**,**B**), metabolic activity using an MTT assay (**C**,**D**), and DNA synthesis using the BrdU assay (**E**,**F**). Results are presented as mean ± SEM of at least 4 measurements. * *p* < 0.05 vs. control; ^ *p* < 0.05 colon cells treated with both NK-92 cells and YGB extract vs. colon cells treated with only NK-92 cells; # *p* < 0.05 colon cells treated with both NK-92 cells and YGB extract vs. colon cells exposed to YGB at the same concentration as those being tested in a one-way ANOVA test; Tukey post hoc test.

Before studying the impact of YGB on NK cells’ cytotoxic activity, the extract’s influence on normal and cancer colon cells was examined via LDH, MTT, and BrdU assays. As presented in Figure 5 (left panel), YGB in the whole range of analyzed concentrations (from 50 to 1000 µg/mL) did not affect the viability of the human colon epithelial cells (CCD841 CoN), which was seen in all the performed assays. On the contrary, when tested at the highest concentration, the extract decreased the metabolic activity of human colon adenocarcinoma LS180 cells by 21.3% (Figure 5D), and simultaneously inhibited DNA synthesis in the investigated cell line by 10.7% (Figure 5F). At the same time, when tested at concentrations of 50, 250, and 1000 µg/mL YGB, the extract was cytotoxic against colon cancer cells, as was shown by the increase in LDH release to the culture medium by 26.5%, 31.8%, and 36.4%, respectively (Figure 5D).

In the next step, YGB’s influence on the NK-92 cells’ cytotoxicity was determined under the same conditions. First, NK-92 cells, when used alone, did not cause any side effects in the investigated human colon epithelial cells. Similarly, the NK-92 cells’ impact on CCD841 CoN viability did not change in the presence of tested extract (Figure 5; left panel). On the contrary, NK-92 cells distinctly inhibited the metabolic activity and DNA synthesis of human colon cancer cells and disrupted the integrity of their cell membranes. Furthermore, the anticancer properties of NK-92 cells were enhanced by YGB in a dose-dependent manner. As presented in Figure 5B, the cytotoxic effect of NK-92 cells in response to treatment with YGB increased from 133.9% to 143.5% (50 µg/mL), 147.3% (250 µg/mL), and 162.5% (1000 µg/mL). The beneficial effect of tested compounds was also observed in another assay. The MTT test (Figure 5D) revealed that the metabolic activity of colon cancer cells treated with both NK-92 cells and the tested extract decreased from 74.7% to 54.9% (50 µg/mL), 51.0% (250 µg/mL), and 47.5% (1000 µg/mL), while the BrdU assay (Figure 5F) showed the significant antiproliferative properties of NK cells against LS180 cells, which intensified in the presence of YGB, as reflected in the decrease in DNA synthesis from 85.5% to 66.5% (50 µg/mL), 62.8% (250 µg/mL), and 59.5% (1000 µg/mL). It should be highlighted that the inhibition of the LS180 cells’ metabolic activity, as well as the DNA synthesis caused by NK-92 in the presence of YGB in the whole range of tested concentrations, was significantly stronger than the decrease in cancer cell viability induced by the extract when used alone (comparison of corresponding YGB concentrations). A similar pattern of changes was observed in the results of the LDH test. Nevertheless, a comparison of results obtained from LS180 cells treated with only NK-92 cells, vs. LS180 exposed simultaneously to both NK cells and YGB, revealed statistically significant differences in cancer cell viability and proliferation at the whole range of tested extract concentrations, while a significant distortion of cell membrane integrity was only noted at 1000 µg/mL YGB.

### 2.4. Young Green Barley Extract Enhances Colon Cancer Cell Death in Response to NK-92 Cells

**Figure 6:**
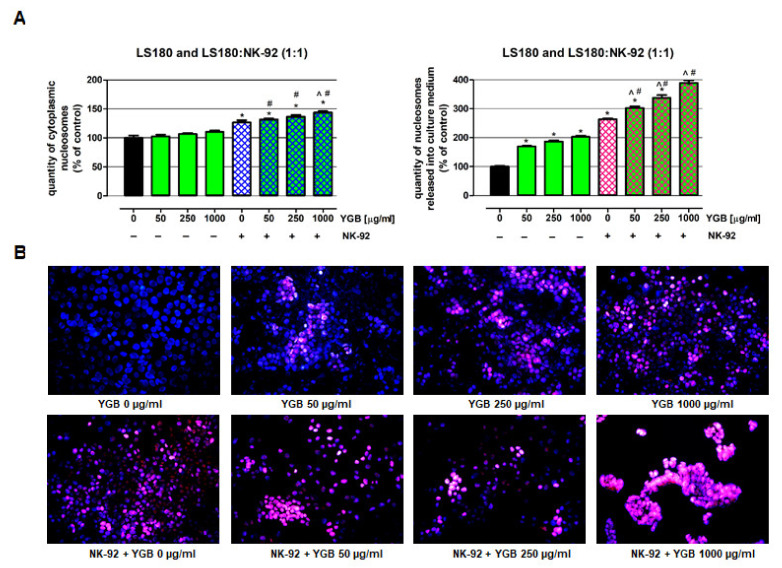
Cell death induction in human colon cancer cells, in response to NK cells co-incubated with young green barley extract (YGB). The impact of YGB (50, 250, and 1000 µg/mL) or NK-92 cells, used together or separately, on the viability of the human colon adenocarcinoma cell line LS180 was investigated after 48 h of incubation. (**A**) Enrichment of nucleosomes in the cytoplasmic fraction (an apoptosis marker) or cell culture medium (a necrosis marker) was determined by a cell-death detection ELISA assay. The results are presented as the mean ± SEM of at least 4 measurements. * *p* < 0.05 vs. control; ^ *p* < 0.05 cancer cells treated with both NK-92 cells and YGB extract vs. cancer cells treated with only NK-92 cells; # *p* < 0.05 cancer cells treated with both NK-92 cells and YGB extract vs. cancer cells exposed to YGB at the same concentration as tested. One-way ANOVA test; post hoc test: Tukey. (**B**) Untreated LS180 cells (control), as well as LS180 cells exposed to the extract with or without NK-92 cells, were double-stained (Hoechst and propidium iodide) and examined under fluorescence microscopy. Representative pictures are presented; the magnification is 400×.

To confirm the observed cytotoxic effect in LS180, cells of NK-92, used separately and together, a YGB cell death detection ELISA assay (Figure 6A) and nuclear double staining were performed (Figure 6B). The results of the ELISA assay revealed that the investigated extract in the tested concentrations was not able to induce apoptosis in LS180 cells at the same time as a number of nucleosomes were released into the cell culture medium (a marker of necrosis) and increased by 69.9% (50 µg/mL), 86.5% (250 µg/mL), and 103.2% (1000 µg/mL). NK-92 cells, when used alone, killed LS180 cells and induced both programmed cell death and necrosis, which was demonstrated by the increase in the number of DNA fragments recorded in the cytoplasmic fraction of colon cancer cells (126.5% of control) and the medium collected from above the cell culture (264.2% of control). Similar to the MTT and LDH results, this research revealed that the anticancer properties of NK-92 cells were enhanced by YGB in a dose-dependent manner. The proapoptotic abilities of NK-92 cells in LS180 cells increased in response to YGB, from 126.5% (LS180 cells treated with only NK-92 cells) to 131.8% (50 µg/mL), 136.4% (250 µg/mL), and 143.5% (1000 µg/mL); however, the statistically significant enhancement of NK-92 cells’ ability to induce programmed cell death was observed only after NK cells were co-incubated with 1000 µg/mL YGB. Conversely, the number of necrotic colon cancer cells that were observed in response to NK-92 when co-incubated with the tested extract increased from 264.2% (LS180 cells treated with only NK-92 cells) to 302.9% (50 µg/mL), 337.6% (250 µg/mL), 388.8% (1000 µg/mL), and the reported YGB intensification of NK cells’ cytotoxicity was statistically significant in the whole range of the investigated extract concentrations. Furthermore, LS180 cell-killing by NK-92 in the presence of YGB, in the whole range of tested concentrations, was significantly improved compared to the direct anticancer effect of the extract (comparison of corresponding YGB concentrations).

The results described above were reflected in the microscopic analysis of LS180 cells that were double-stained with Hoechst and propidium iodide (Figure 6B). The conducted studies have shown single colon cancer cells undergo programmed cell death in both untreated and YGB-treated LS180 cells. Nevertheless, YGB, when used alone, significantly damaged colon cancer cell membranes, and the observed cytotoxicity was dose-dependent. On the contrary, NK-92 cells induced both necrosis and apoptosis in the investigated colon cancer cells; however, the cytotoxic effect was dominant. The anticancer effect of the NK cells distinctly increased in the presence of the tested extract, which was associated with elevated levels of both necrotic and apoptotic LS180 cells; however, the disintegration of cell membranes was the dominant mode of cancer cell elimination. The enhancement of colon cancer cell death by YGB-treated NK-92 cells was directly dependent on extract concentration.

## 3. Discussion

Young green barley water extract has been shown to contain more than 64% of saccharides, composed mainly of glucose and fructose. Methylation analysis shows that the majority of them are in the terminal position, which might suggest that the preparation contains short oligosaccharides. Interestingly, the presence of saccharose—the metabolic precursor of fructans—could not be observed in the MALDI-TOF mass spectrum. This means that saccharose can be quickly metabolized or exported outside of the leaves. Fructans are considered to be the main saccharide reserves in the vegetative tissues of grasses, including barley [26]. The oligosaccharides observed in the analyzed extract can be recognized as plant fructan. Mixed-type fructan (containing both (1→2) and (6→2)-linked fructosyl units were found in the YGB preparation, based on methylation analysis and the mass spectrometry technique. The fructan content in YGB was also determined and represented approximately 10.2% of the dry matter basis of the analyzed samples. This type of sugar polymer is typical for cereals, such as barley [27,28]. Plant fructans are usually shorter than bacterial ones and, in general, contain less than 50 fructosyl units. Nemeth and co-workers showed changes in the distribution of different chain lengths and the pattern of fructan structures in the barleys derived from different cultivars or breeding lines [29]. In the case of YGB extract, the fructose oligomers had fewer than 20 units, and the most frequent fraction that was found contained 4 sugars—this is probably nystose. Nystose was described earlier as a component of barley grains [30].

Despite the fact that the immunomodulatory properties of plant fructans are becoming better and better known, most of the studies focused only on their indirect influence on the immune system components, which is a consequence of their prebiotic properties [31,32]. Nevertheless, the results from recent studies dedicated to fructans isolated from a wide range of plants revealed that the mentioned carbohydrates may also directly impact immune cells and, consequently, modulate the immune system responses [31]. Unfortunately, there is a lack of evidence presenting the direct impact of barley fructans on the components of the immune system. Nevertheless, considering the fact that the main component of the investigated extract was also fructooligosaccharide, the discovery of a beneficial impact on immune cells was predicted.

It should be emphasized that the chemical examination of YGB revealed that, next to carbohydrates, the second main component of the investigated extract was proteins, the total amount of which was around 27%. The electrophoretic separation of young green barley extract revealed the presence of proteins with a molecular weight of around 55 kDa and 35 kDa, as well as proteins weighing less than or equal to 15 kDa. The obtained data indicated that YGB was a carbohydrate–protein complex. Considering the proven enhancement impact of protein ligands on both the anticancer and immunomodulatory properties of polysaccharides [33], discovered that the presence of proteins in YGB seems to be beneficial. It should be emphasized that, according to our best knowledge, the data presented herein is the first report published about the immunomodulatory properties of the barley carbohydrate-protein complex.

The immunomodulatory properties of young green barley extract were examined using NK-92 cells, the best-described human NK cell line, the therapeutic utility of which has been confirmed in several clinical trials [34,35]. NK-92 cells characterized the ability to produce cytokines, as well as high cytotoxic activity. Although they are highly cancer-specific, the cells can be further modified to express different cancer receptors to increase the targeting and killing of cancer cells, as well as to express therapeutic antibody-binding receptors, which also enhances their anticancer activity. Furthermore, NK-92 cells are able to resist the suppression induced by many agents released by cancer cells, thanks to the absence of killer inhibitory receptors (KIR) [35,36]. The above-mentioned features, as well as their long-term growth potential, have made NK-92 cells an attractive model by which to study the function of human NK cells, and have become an excellent tool for evaluating the immunoenhancement properties of compounds of various origins; thus, this cell line was selected to examine the possibility of using polysaccharide-rich young green barley extract in oncological immunotherapy.

Evaluation of the direct impact of young green barley extract on NK cells revealed that the tested compounds are not cytotoxic in the whole range of investigated concentrations, and did not affect the viability and proliferation of NK-92 cells in a wide range of tested concentrations (from 25 to 250 μg/mL); however, at higher doses (500 and 1000 μg/mL) the extract decreased both lymphocyte metabolic activity and DNA synthesis. Several studies have reported the stimulation of leucocyte proliferation by barley polysaccharides; however, the beneficial effect on NK cell numbers was reported only by Han et al., who revealed the increase of NK cell proliferation in immunosuppressive BALB/c mice in response to water-soluble barley polysaccharide (BP-1; molecular weight 67 kDa) [11]. Nevertheless, it needs to be highlighted that NK-92 cells are human lymphoma-derived cells and, despite the fact that they are phenotypically similar to activated normal NK cells [36], the results obtained from studies conducted on this cell line should be interpreted in light of this data. Because of the reaction observed in response to higher doses of young green barley extract, the slight decrease of NK-92 cell viability and proliferation with a simultaneous lack of YGB cytotoxicity did not unequivocally determine the existence or absence of the immunomodulatory properties of the test substance.

In order to verify the possibility of using young green barley extract-derived carbohydrate-protein complex in oncological immunotherapy based on NK cells, YGB’s influence on NK-92 anticancer activity was evaluated. Because immunotherapy should be both effective and safe, studies have been conducted on both normal and cancer cell lines (human colon epithelial CCD841 CoN cells and human colon cancer LS180 cells). An in vitro model of colon cancer was chosen because of the fact that this type of cancer is one of the most common cancers in terms of incidence and mortality rate in the world.

In the first step of these studies, the direct anticancer effect of YGB as well as NK cells used separately has been analyzed. The obtained results revealed that the examined compounds significantly decreased the viability and proliferation of colon cancer LS180 cells, as well as effectively damaging their cell membranes and causing necrosis induction. At the same time, YGB did not affect human colon epithelial CCD841 CoN cells. The observed high selectivity of the examined compounds can be understood as a lack of negative influence on normal colon cells, with an evident anti-cancer effect against colon cancer cells, which corresponds with the previous results of our research group [37,38].

Similar to YGB, NK-92 cells also revealed a high selectivity of action in relation to colon cancer cells. The recorded anticancer effect of NK cells was mostly associated with cytotoxicity; however, significant apoptosis induction was also noted. In terms of oncological immunotherapy, the observed ability of NK-92 cells to induce different cancer cell death would create the possibility of modulating the immunogenicity of the tumor microenvironment, which is important in the case of therapy efficiency and safety.

The discovered anticancer effect of NK-92 cells and YGB, when used alone, intensified when the lymphocytes and the extract were used together. The beneficial impact of NK cells co-incubated with the examined polysaccharide-rich young green barley extract was observed on metabolic activity, DNA synthesis, cell membrane integrity, and cell death induction in LS180 cells. The enhancement of NK cells’ anticancer activity by YGB was dose-dependent; nevertheless, a significant improvement in the NK-92 cells’ proapoptotic and cytotoxic abilities was noted only in the presence of tested compounds at the highest tested concentration (1000 μg/mL), while other investigated phenomena were evidently improved by YGB in the whole range of tested concentrations. It should be highlighted that cancer cell elimination by NK-92 in the presence of YGB in the whole range of tested concentrations was significantly stronger than the decrease in cancer cell viability and proliferation, as induced by the extract when used alone at the corresponding concentrations. Nevertheless, the synergy of the anticancer actions of NK-92 cells and the tested compounds was observed in all performed studies except the LDH assay. The YGB in the whole range of investigated concentrations that were given to cancer cells together with NK cells caused a stronger inhibition of metabolic activity and DNA synthesis, as well as improved apoptosis induction, than would have been anticipated from the mere summation of the direct effects of the lymphocytes and tested compounds. In the case of necrosis, a synergy of action was observed in an NK cell co-incubated with YGB at a concentration of 1000 μg/mL. The observed enhancement of the NK cells’ cytotoxic abilities in response to barley polysaccharides corresponds with previously published data [10]. Ex vivo studies conducted by Han et al. revealed that the oral administration of crude barley polysaccharides (BLE0; mainly composed of neutral sugars and uronic acid and containing minor levels of 2-keto-3-deoxy-mannooctanoic acid-like materials) significantly recovered the cyclophosphamide-induced reduction of NK cell activity against YAC-1 mouse T cell lymphoma cells [10].

In summary, the results of the present studies indicate that polysaccharide-rich young green barley extract may have immunomodulatory properties associated with the enhancement of NK cells’ ability to recognize and eliminate human colon cancer cells without any side effects on normal colon epithelial cells. Furthermore, the obtained data also revealed the direct anticancer effect of the tested compounds, as well as high selectivity against neoplastic cells. Although the presented studies were performed only on cell lines, their importance is supported by the fact that they focused on NK92 cells with proven therapeutic utility in clinical studies. The discovered beneficial impact of the investigated extract on NK92 cells’ anticancer properties brings hope for its therapeutic use, especially as an adjuvant in the currently applied immunotherapies for colon cancers. Despite the fact that over 90% of the extract components have been determined, further study is necessary to recognize other functional ingredients, next to the discovered carbohydrate-protein complexes, that may also influence the discovered immunoenhancement properties of YGB.

## 4. Materials and Methods

### 4.1. Reagents

Unless indicated otherwise, the chemicals used in this study were purchased from Sigma-Aldrich Co. LLC, Saint Louis, MO, USA.

### 4.2. Preparation of Young Green Barley Extract

The powdered young green barley juice (*Hordeum vulgare*) was purchased from Green Ways (Prague, Czech Republic). First, 5 g of the product was dissolved in 150 mL sterile water. Extraction was then carried out for 24 h on a rotator at room temperature. The resulting mixture was centrifuged (4075× *g*, 10 min, 20 °C) and the collected supernatant was filtered through a microbiological filter. The resulting filtrates were then subjected to a freeze-drying process. The lyophilizate thus obtained was stored at −20 °C. The stock solution of young green barley (25 mg/mL) was prepared by dissolving the lyophilizate in PBS (phosphate-buffered saline). The stock solution was stored at −20 °C. Working solutions of the extract were prepared by dissolving a stock solution in a culture medium. In this manuscript, young green barley extract has been expressed as YGB.

### 4.3. Sugar Content Determination

The total sugar content in YGB was determined using the phenol-sulphuric acid method, as previously described [39], using glucose as a standard. The tested extract (200 µL) was mixed with 5% aqueous phenol solution (200 µL) and concentrated H_2_SO_4_ (1 mL). After 10 min of incubation at room temperature, absorbance was recorded on a microplate reader, the BioTek ELx800 (Highland Park, Winooski, VT, USA) at 490 nm wavelength. The results were expressed as the percentage of YGB.

### 4.4. Protein Content Determination

The protein concentration in YGB was determined using a Pierce BCA Protein Assay Kit (Thermo Fisher Scientific, Waltham, MA, USA) following the manufacturer’s instructions. Bovine serum albumin was used as a standard. Absorbance was recorded on a microplate reader, a BioTek ELx800, at 570 nm wavelength. The results were expressed as the percentage of YGB.

### 4.5. Proteins Separation—SDS-PAGE

YGB at a concentration of 25 mg/mL was solubilized in sample buffer (30% glycerol, 10% SDS, 0.5 M Tris-HCl, pH 6.8, 0.012% bromophenol blue, 5% β-mercaptoethanol) and boiled for 5 min. Then, 10 μL of the YGB sample, as well as 5 μL of Page Ruler Plus pre-stained protein ladder (Thermo Fisher Scientific, Waltham, MA, USA), were loaded onto the 12% polyacrylamide gel. Protein separation was carried out at 80 V for 30 min, followed by 200 V for 60 min for the resolving gel, using a Power Pac (Bio-Rad Laboratories, Hercules, CA, USA). The gel was stained for 30 min with Coomassie Brilliant Blue, dissolved in water/methanol/acetic acid (5/4/1, *v/v/v*), and the destaining of the gel was achieved using Coomassie solvent for 3 h. The estimation of the molecular mass of proteins was performed using the abovementioned molecular protein mass marker (from 15 to 250 kDa).

### 4.6. Chemical Analyses of Sugars

Monosugars were liberated from 1 mg of the YGB sample by hydrolysis, using 2 M trifluoroacetic acid (100 °C, 4 h), and were converted into alditol acetates via reduction with NaBD_4_ and acetylation, as described elsewhere [40]. The obtained alditol acetates were analyzed via gas-liquid chromatography coupled with mass spectrometry (GLC-MS) technique.

To obtain the acetylated methyl glycosides, the YGB sample was subjected to methanolysis (2 M HCl/methanol, 85 °C, 2 h) and acetylation (pyridine/acetic anhydride, 85 °C, 30 min).

To obtain the peracetylated derivatives of methyl oximes, the YGB preparation was mildly hydrolyzed (1 M HCl_aq_ 50 °C, 1 h) and dried under a vacuum. Methoxylamine hydrochloride (100 µL, 0.18 M in pyridine) was added and the sample was incubated at 70 °C for 1 h. Acetic anhydride was added (100 µL), then a sample was incubated at 45 °C for 1 h. The sample was dried, dissolved in ethyl acetate, and analyzed by GLC-MS. The standard aldoses (mannose, glucose, galactose) and ketoses (fructose, tagatose, sorbose) were prepared in the same way.

The linkage position between sugar components in the sample was established by methylation analysis [41] and the extraction of permethylated products into chloroform, followed by acid hydrolysis (2 M TFA, 100 °C, 4 h), reduction with NaBD_4_, and acetylation. The obtained partly methylated alditol acetates were analyzed by GLC–MS.

### 4.7. Fructan Content Determination

The fructan content was determined using the PAHBAH reducing sugar method, with the help of the Fructan Assay Procecedure Kit (Megazyme, Bray, Ireland), following the manufacturer’s instructions. First, 100 mg increments of three independent lyophilized samples were used in triplicate. The PAHBAH color complex absorbance was recorded on a microplate reader, BioTek ELx800, at a 405 nm wavelength. Fructan content was calculated using the Megazyme Mega-Calc spreadsheet (Megazyme, Ireland).

### 4.8. FTIR Spectroscopy

An infrared absorption spectrum (FTIR-ATR) of the YGB was recorded using an FTIR spectrometer, the Nicolet 8700A (Thermo Scientific). The spectral range was from 400 to 4000 cm^−1^.

### 4.9. GLC-MS Analysis

The sugar derivatives were analyzed by GLC-MS using a gas chromatograph 7890A (Agilent Technologies, Inc., Wilmington, DE, USA) connected to a mass selective detector (MSD 5975C, inert XL EI/CI (Agilent Technologies, Inc., Wilmington, DE, USA)). The chromatograph was equipped with an HP-5MS column (30 m × 0.25 mm) and used helium as a carrier gas. The temperature program was as follows: 150 °C for 5 min, increased to 310 °C (5 °C min^−1^); the final temperature was maintained for 10 min. The components were identified based on their mass spectra and retention time and then compared with the known standards.

### 4.10. Mass Spectrometry

The MALDI-TOF MS (Matrix-Assisted Laser Desorption/Ionization—Time of Flight Mass Spectrometry) and MS-MS (tandem mass spectrometry) of YGB were performed with a SYNAPT G2-Si HDMS instrument (Waters Corporation, Milford, MA, USA) equipped with a 1 kHz Nd: YAG laser system (355 nm wavelength). Acquisition of the data was performed using the MassLynx software, version 4.1 SCN916 (Waters Corporation, Wilmslow, UK). To act as a matrix, 2,5-dihydroxybenzoic acid solution (20 μg/μL) in 50% acetonitrile was used. The sample was dissolved in 50% methanol to a concentration of 20 μg/μL and then mixed with matrix solution in the proportion of 1:1 (*v*/*v*) [42]. Spectra were recorded in a positive ion mode. For MS/MS experiments, isolated precursor ions were fragmented using a collision voltage of 15 V. MS data were collected for 120 s. Mass spectra were assigned with multi-point external calibration using red phosphorous (Sigma Aldrich, St. Louis, MO, USA).

### 4.11. Cell Lines

Human natural killer cells (NK-92) and human colon epithelial cells (CCD841 CoN) were obtained from the American Type Culture Collection (ATCC, Manassas, VA, USA). Human colon adenocarcinoma cells (LS180) were obtained from the European Collection of Cell Cultures (ECACC, Center for Applied Microbiology and Research, Salisbury, UK).

NK-92 cells were grown in Alpha Minimum Essential Medium without ribonucleosides and deoxyribonucleosides, and with 2 mM L-glutamine and 1.5 g/L sodium bicarbonate, supplemented with 0.2 mM inositol, 0.1 mM 2-mercaptoethanol, 0.02 mM folic acid, 200 U/mL recombinant IL-2, 12.5% horse serum, and 12.5% fetal bovine serum (FBS). CCD841 CoN cells were grown in Dulbecco’s Modified Eagle’s Medium (DMEM) supplemented with 10% FBS. LS180 cells were grown in a 1:1 mixture of DMEM and nutrient mixture, Ham F-12, supplemented with 10% FBS. All media were supplemented with penicillin (100 U/mL) and streptomycin (100 μg/mL). Cells were maintained in a humidified atmosphere of 95% air and 5% CO_2_ at 37 °C.

### 4.12. Cell Treatment

In the case of the MTT, LDH, and BrdU assays, cells were seeded on 96-well microplates at a density of 5 × 10^4^ cells/mL. In the case of microscope evaluation, cells were seeded on Lab-Tek Chambers Slides (Nunc) at a density of 5 × 10^4^ cells/mL.

Basic variant: Immediately after seeding on cell culture vessels, the NK-92 cells were exposed to a series of YGB dilutions. Conversely, adherent cells (CCD841 CoN, LS180) were treated with a series of YGB dilutions 24 h after cell seeding into cell culture vessels. YGB dilutions were prepared in a culture medium suitable for individual cell lines.

The variant in co-cultures: Adherent cells were seeded on cell culture vessels. The following day, the culture medium was removed, and the cells were exposed to NK92 cells in the absence or presence of a series of YGB dilutions. The proportion of adherent cells to NK cells was 1:1. The YGB dilutions were prepared in a culture medium suitable for NK-92 cells.

In both variants of the experiments, cells were treated with YGB, or NK-92 cells, or YGB + NK-92 cells for 48 h, and afterward, proper investigations were performed.

### 4.13. Cytotoxicity Assessment—LDH Assay

After treatment, the cells growing on microplates were centrifuged at 300× *g* for 10 min, at room temperature. Then, the culture supernatants were collected in new 96-well microplates, which were used to perform an LDH assay following the manufacturer’s instructions (In Vitro Toxicology Assay Kit—Lactate Dehydrogenase-Based, Sigma Aldrich, St. Louis, MO, USA). Absorbance was recorded on a microplate reader (BioTek ELx800), at a wavelength of 450 nm.

### 4.14. Assessment of Metabolic Activity—MTT Assay

After cell treatment, the metabolic activity of cells was investigated using the MTT test. In brief, cells were incubated with MTT solution (5 mg/mL in PBS) for 6 h. Then, formazan crystals were solubilized overnight in SDS buffer (pH 7.4) (10% SDS in 0.01 N HCl), and the product was quantified spectrophotometrically by measuring the absorbance at 570 nm wavelength using the microplate reader (BioTek ELx800).

### 4.15. Assessment of DNA Synthesis—BrdU Assay

After cell treatment, DNA synthesis was measured with a colorimetric immunoassay, Cell Proliferation ELISA BrdU (Roche Diagnostics GmbH, Penzberg, Germany), according to the manufacturer’s instructions. Absorbance was recorded on a microplate reader (BioTek ELx800) at a wavelength of 450 nm.

### 4.16. Cell Death Detection—ELISA

After cell treatment, cell death (both apoptosis and necrosis) was assessed using the Cell Death Detection ELISA PLUS kit (Roche Diagnostics, Mannheim, Germany), according to the manufacturer’s instructions. Studies were conducted in cytoplasmic fractions as well as the cell medium collected from above the cell cultures, which allowed the determining of nucleosome quantity in apoptotic and necrotic cells, respectively. Absorbance was measured at a wavelength of 405 nm, using a microplate reader (BioTek ELx800).

### 4.17. Cell Death Detection—Nuclear Double Staining

After treatment, cell death was visualized using nuclear double-staining. Cells were incubated for 5 min with a fluorochrome mixture: Hoechst 33342 (0.24 mg/mL) and propidium iodide (PI) (0.15 mg/mL). The stained cells were observed under an Olympus BX51 System microscope and the micrographs were prepared using the CellFamily AnalySIS software.

### 4.18. Statistical Analysis

The obtained data were developed in the following programs: Microsoft Excel 2010 and GraphPad Prism 5.0. The results are presented as the mean value and standard error of the mean (SEM). The data were analyzed using a one-way ANOVA test with Dunnett’s or Tukey’s post hoc tests, and column statistics were used for comparisons. Significance was accepted at *p* < 0.05.

## Figures and Tables

**Table 1:** Linkage analysis of young green barley extract (YGB). The sample was subjected to methylation, hydrolysis, reduction, and acetylation. The obtained permethylated alditol acetates were identified by GLC–MS, based on their mass spectra and retention times. The main permethylated alditol acetates are shown in bold type.

Component	Amount (%)
***t*** **-Fru*f*-(2** **→**	**22.3**
*t*-Man*p*-(1→	1.2
***t*** **-Glc*p*-(1** **→**	**47.2**
*t*-Gal*p*-(1→	5
**→** **6)-Fru*f*-(2** **→**	**9.7**
→3)-Glc*p*-(1→	0.9
→4)-Glc*p*-(1→	3.1
→3)-Gal*p*-(1→	0.5
→6)-Glc*p*-(1→	3
→6)-Gal*p*-(1→	2
→1,6)-Fru*f*-(2→	3.5
→3,6)-Glc*p*-(1→	1.6

## Data Availability

The data presented in this study are available on request from the corresponding author. The data are not publicly available due to privacy.

## Sample Availability

Samples of the investigated extract are available from the authors.
